# Coactosin‐Like Protein Reduces Prostaglandin D_2_ Production in Alveolar Macrophages and Alleviates Allergic Airway Inflammation

**DOI:** 10.1002/advs.202501673

**Published:** 2025-06-26

**Authors:** Li‐Long Pan, Zhengnan Ren, Binbin Li, Wenjie Liang, Yang Luo, Qin Yang, He Liu, Xiaoliang Dong, Haizhi Tian, Huimin Zou, Bengt Samuelsson, Olof Rådmark, Jia Sun

**Affiliations:** ^1^ School of Food Science and Technology Wuxi School of Medicine Jiangnan University Wuxi Jiangsu 214122 China; ^2^ MOE Medical Basic Research Innovation Center for Gut Microbiota and Chronic Diseases Jiangnan University Wuxi Jiangsu 214122 China; ^3^ State Key Laboratory of Food Science and Resources Jiangnan University Wuxi Jiangsu 214122 China; ^4^ Department of Medical Biochemistry and Biophysics Division of Physiological Chemistry II Karolinska Institutet Stockholm 17177 Sweden

**Keywords:** allergic asthma, alveolar macrophage polarization, chemoattractant receptor‐homologous molecule expressed on Th2 cells, coactosin‐like protein, prostaglandin D_2_

## Abstract

Allergic asthma is a significant global health issue characterized by chronic airway inflammation. Current treatments only alleviate symptoms but fail to cure the disease due to its complex pathology. Lipid mediators from arachidonate metabolism are pivotal in immune regulation in asthma. Previously, coactosin‐like protein (CLP) is identified as a regulator of leukotriene production in vitro. However, its role in asthma is unclear. In this study, it is found that CLP‐deficient (*Cotl1* ^−/−^) mice challenged with house dust mite (HDM) exhibits exacerbated airway inflammation, macrophage polarization, and type 2 immune responses. CLP deficiency increased prostaglandin D_2_ (PGD_2_) in bronchoalveolar lavage (BAL) and alveolar macrophages (AMs), activating the PGD_2_ receptor chemoattractant receptor‐homologous molecule expressed on Th2 cells (CRTH2) on immune cells. Notably, HDM exposure reduced pulmonary CLP levels in wild‐type (WT) mice, and overexpression of CLP in *Cotl1^−/−^* macrophages decreased HDM‐induced PGD_2_ in BAL and alleviated inflammation. *Cotl1^−/−^* AMs exacerbated HDM‐induced airway inflammation compared to WT AMs, and this effect is dependent on CRTH2 signaling. These findings reveal that CLP modulates macrophage polarization and suppresses the PGD_2_‐CRTH2 pathway to alleviate airway inflammation, highlighting CLP as a promising therapeutic target for asthma.

## Graphical abstract

Coactosin‐like protein inhibits prostaglandin D₂ (PGD_2_) secretion by modulating alveolar macrophage polarization, thereby suppressing activation of the PGD_2_ receptor CRTH2 in Th2‐associated immune cells and alleviating allergic airway inflammation.

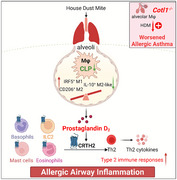

## 1 Introduction

Allergic asthma is a T helper 2 (Th2)‐dependent chronic airway inflammation associated with airway remodeling.^[^ ^1^, ^2^ ^]^ The prevalence of allergic asthma has significantly increased in recent years, imposing a substantial global health and economic burden. Anti‐inflammatory drugs such as inhaled corticosteroids, being the mainstay therapy for allergic asthma, have been reported to cause adverse reactions, including adrenal suppression, reduction in growth velocity, metabolic changes, and behavioral abnormalities. Furthermore, a large number of patients treated with inhaled corticosteroids remain symptomatic.^[^ ^3^, ^4^, ^5^ ^]^ Arachidonic acid (AA) metabolism is central to allergic asthma pathophysiology due to the production of a spectrum of bioactive mediators, such as prostaglandins (PGs).^[^ ^6^ ^]^ In allergic asthma, prostaglandin D_2_ (PGD_2_), a major prostanoid, contributes to the disease via its receptor chemoattractant receptor‐homologous molecule expressed on Th2 cells (CRTH2).^[^ ^7^, ^8^, ^9^, ^10^ ^]^ Moreover, CRTH2‐mediated signals enhance the production of interleukin (IL) alveolar macrophages (‐4, IL‐5 and IL‐13 by Th2 cells.^[^ ^11^ ^]^ However, effective drugs targeting these mediators remain limited. Therefore, identifying and targeting key proteins involved in AA metabolism may provide novel strategies for the prevention of allergic asthma.

Coactosin‐like protein (CLP) is a member of the actin‐depolymerizing factor‐homology family,^[^ ^12^ ^]^ regulating actin dynamics in various cells.^[^ ^13^, ^14^ ^]^ Our previous work has shown that CLP binds to 5‐lipoxygenase (5‐LO), serving as a stabilizing scaffold for increased enzyme activity, thereby influencing AA metabolism.^[^ ^15^, ^16^, ^17^ ^]^ In monocytic cells, CLP knockdown reduces leukotriene (LT) formation and inhibits 5‐LO translocation from the cytosol to the nuclear membrane.^[^ ^18^ ^]^ A recent study showed that CLP is downregulated in the airway mucus of asthma patients compared with healthy controls.^[^ ^19^ ^]^ These studies suggest that CLP may be involved in the pathogenesis of airway inflammation by regulating the balance of the AA pathway. However, the effect of CLP on allergic airway inflammation in vivo remains uncertain. To address this question, we generated a CLP‐deficient mouse model and exposed it to house dust mites (HDM). Our studies highlight the potential of CLP as a promising therapeutic target for asthma.

## 2 Results

### 2.1 Dysregulated CLP Expression Contributes to Susceptibility to HDM‐Induced Airway Inflammation

**Figure 1:**
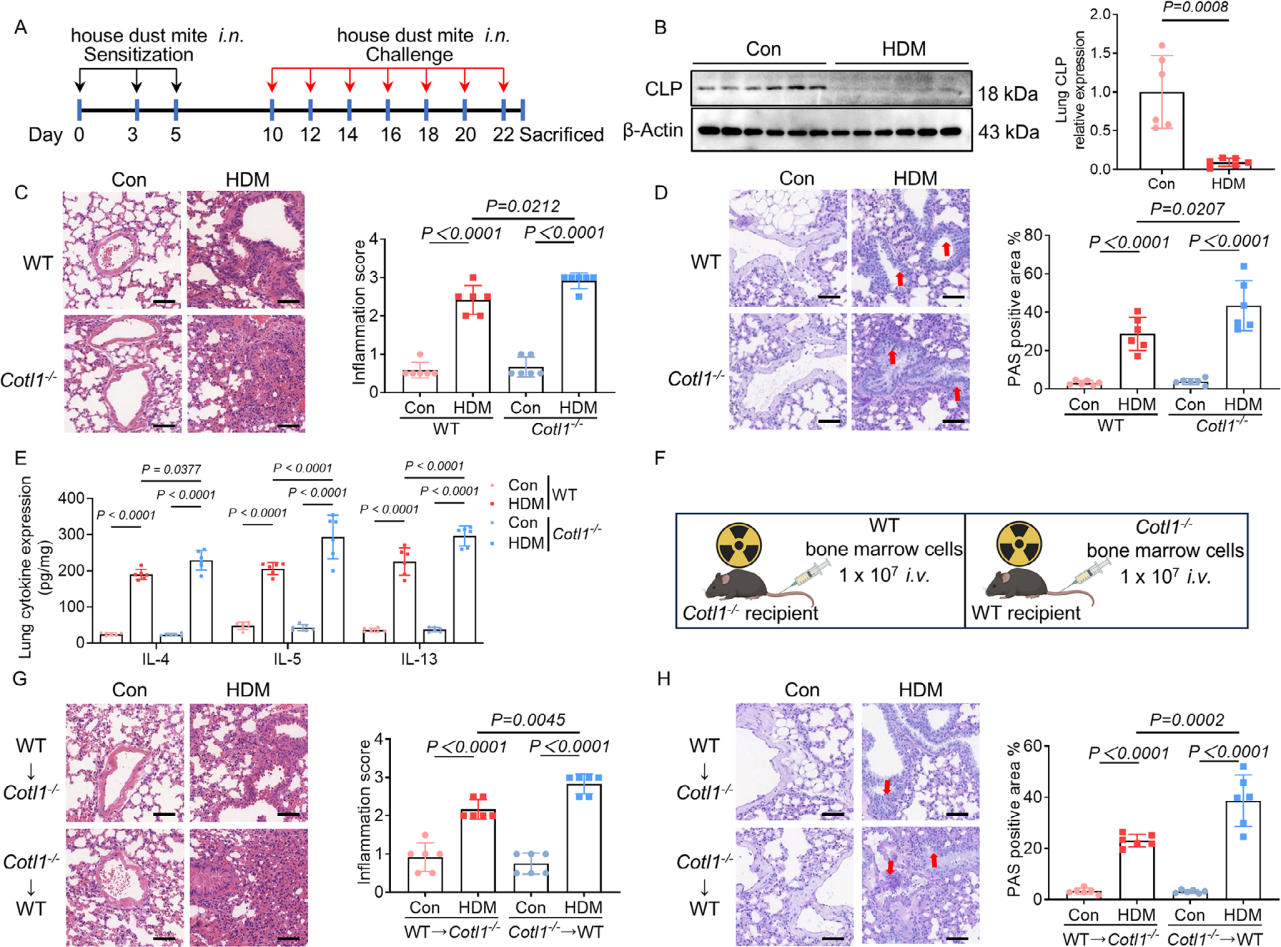
House dust mite allergen induces more severe bronchial inflammation in *Cotl1* ^−/−^mice. A) Mice were sensitized and challenged intranasally with either HDM (50 µg) or saline on days 0, 3, 5, 10, 12, 14, 16, 18, 20, 22, and were euthanized on day 23. B) Protein expression of CLP in lung tissue. Western blot analyses were performed on samples from six mice for each condition. C,D) Representative lung samples stained with (C) H&E and (D) PAS (*n* = 6). Scale bar: 50 µm. E) The levels of Th2 cytokines (IL‐4, IL‐5, and IL‐13) in lung tissue extracts were determined by ELISA (*n* = 6). F) *Cotl1^−/−^* mice and WT mice were irradiated and then adoptively transferred with bone marrow‐derived macrophages from WT and *Cotl1^−/−^* mice, respectively. G,H) Representative lung samples stained with (G) H&E and (H) PAS (*n* = 6). Scale bar: 50 µm. Data shown in (B–E), (G), and (H) were presented as mean ± SD. *P* values were assessed using an unpaired two‐tailed *t*‐test for (B), Tukey's multiple comparisons test following one‐way ANOVA for (C), (D), (G), and (H), and Tukey's multiple comparisons test following two‐way ANOVA for (E). *p*< 0.05 was considered statistically significant.

To investigate the role of CLP in regulating HDM‐induced allergic asthma, we examined the protein expression of pulmonary CLP. Compared to control mice, HDM‐sensitized and challenged mice exhibited remarkably reduced CLP expression (**Figure** **1**A,B). Next, we evaluated how CLP deficiency influenced the development of allergic airway inflammation. Intranasal administration of HDM elicited more severe inflammation in CLP‐deficient (*Cotl1* ^−/−^) mice compared to littermate control (wild‐type, WT) mice. Hematoxylin‐eosin (H&E) and periodic acid‐Schiff (PAS) staining of lung sections from HDM‐challenged *Cotl1* ^−/−^ mice revealed increased inflammatory infiltrates and mucus secretion (Figure 1C,D). Additionally, lung tissue from HDM‐challenged *Cotl1* ^−/−^ mice showed higher levels of Th2 cytokines (IL‐4, IL‐5, and IL‐13) compared to WT mice (Figure 1E). To identify which cells contributed to heightened HDM‐induced inflammation in the lungs of *Cotl1* ^−/−^ mice, we generated bone marrow chimeric mice. Recipient WT mice and *Cotl1* ^−/−^ mice were irradiated and reconstituted with bone marrow cells from either *Cotl1* ^−/−^ or WT mice via tail vein injection (Figure 1F). Following the HDM challenge, *Cotl1* ^−/−^→WT mice (irradiated WT mice receiving *Cotl1* ^−/−^ bone marrow) exhibited more severe inflammation and mucus production compared to WT→*Cotl1* ^−/−^ mice (irradiated *Cotl1* ^−/−^mice receiving WT bone marrow) (Figure 1G,H). These data indicate that the elimination of CLP in bone marrow‐derived cells leads to more severe HDM‐induced airway inflammation.

### 2.2 CLP Influences the Polarization of Alveolar Macrophages and the Recruitment of Other Immune Cells

**Figure 2:**
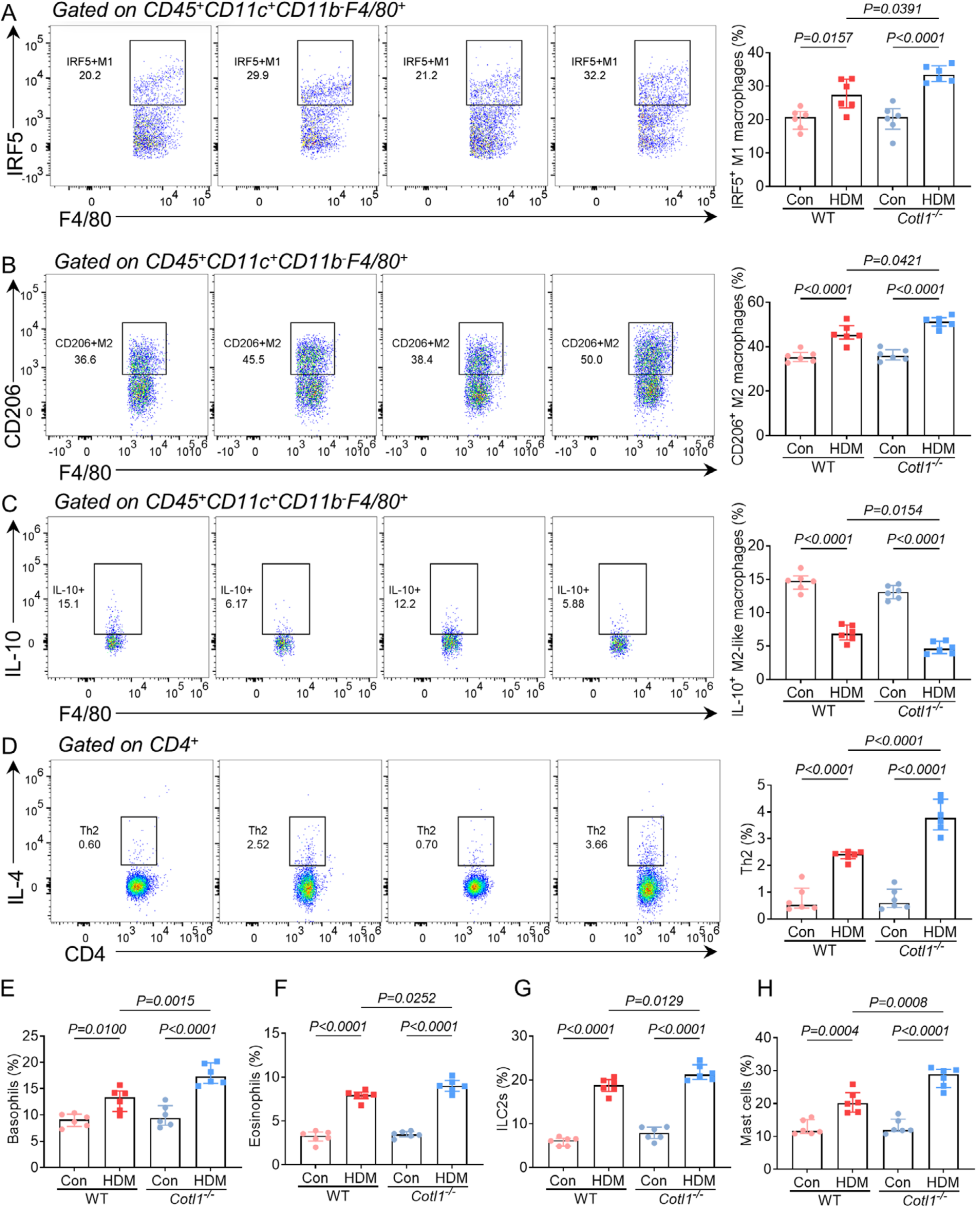
CLP influences the polarization of alveolar macrophages and the infiltration of other immune cells during allergic asthma. On day 23, mice were sacrificed, and lung tissue single‐cell suspensions were prepared and analyzed by flow cytometry. A) Frequencies of IRF5^+^ M1 macrophages among the CD45^+^CD11c^+^CD11b^−^F4/80^+^ population (*n* = 6). B) Frequencies of CD206^+^ M2 macrophages among the CD45^+^CD11c^+^CD11b^−^F4/80^+^ population (*n* = 6). C) Frequencies of IL‐10^+^ M2‐like macrophages among the CD45^+^CD11c^+^CD11b^−^F4/80^+^ population (*n* = 6). D) Frequencies of IL‐4^+^ Th2 cells among the CD4^+^ population (*n* = 6). E) Frequencies of CD49b^+^ basophils among the IgE^+^ population (*n* = 6). F) Frequencies of Siglec‐F^+^ eosinophils among the CD45^+^CD11c^+^ population (*n* = 6). G) Frequencies of Sca‐1^+^KLRG1^+^ ILC2 cells among the CD45^+^CD25^+^Lin^−^ population (*n* = 6). H) Frequencies of CD117^+^ mast cells among the IgE^+^ population (*n* = 6). Data were presented as median ± interquartile range. *P* values were assessed using Tukey's multiple comparisons test following one‐way ANOVA. *p*< 0.05 was considered statistically significant.

Considering the regulatory relationship between the cytoskeleton and macrophage polarization,^[^ ^20^, ^21^ ^]^ we investigated whether CLP knockout could influence the frequencies of different macrophage phenotypes. Using flow cytometry on single‐cell suspensions prepared from the whole lung tissue on day 23, we analyzed the different alveolar macrophage subpopulations after the HDM challenge (**Figure** **2**A–C). Notably, HDM‐challenged *Cotl1* ^−/−^ mice exhibited a higher frequency of IRF5^+^ M1 macrophages compared to WT mice (Figure 2A). Additionally, the frequency of CD206^+^ M2 macrophages increased (Figure 2B), while IL‐10^+^ M2‐like macrophages decreased (Figure 2C) in HDM‐challenged *Cotl1* ^−/−^ mice compared to WT mice. Furthermore, we evaluated the HDM‐induced accumulation of immune cells associated with type 2 immune responses, including Th2 cells, basophils, eosinophils, group 2 innate lymphoid cells (ILC2), and mast cells in lung tissue. All these cell types exhibited increased percentages in HDM‐challenged *Cotl1* ^−/−^ mice compared to WT mice (Figure 2D–H). These results collectively indicate that CLP plays a role in the polarization of alveolar macrophages and the accumulation of key immune cells during airway inflammation.

### 2.3 Increased Formation of PGD_2_ is Observed in *Cotl1^−/−^* BAL and in Alveolar Macrophages

**Figure 3:**
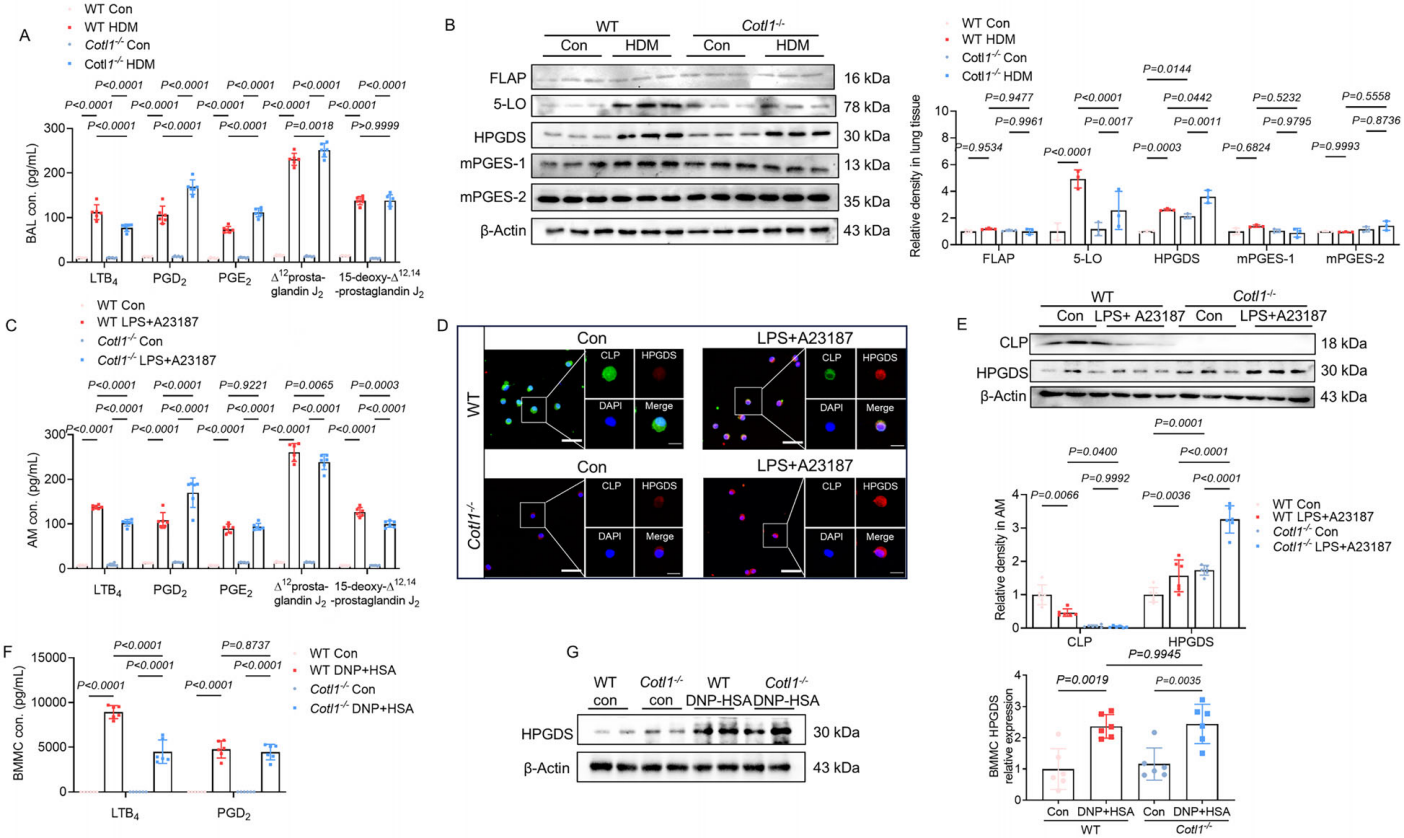
*Cotl1* ^−/−^ mice subjected to HDM challenge have increased PGD_2_ in BAL and AMs. A) Levels of LTB_4_, PGD_2_, PGE_2_, Δ^12^prostaglandin J_2_, and 15‐deoxy‐Δ^12,14^‐prostaglandin J_2_ in BAL fluid (day 23) analyzed by LC‐MS (*n* = 6). B) Expression of FLAP, 5‐LO, HPGDS, mPGES‐1, and mPGES‐2 in lung tissue (day 23). Western blot analyses were performed on samples from three mice for each condition. C) LC‐MS analyses of LTB_4_, PGD_2_, PGE_2_, Δ^12^prostaglandin J_2_, and 15‐deoxy‐Δ^12,14^‐prostaglandin J_2_ present in AMs. Cells were primed with LPS (1 µg·mL^−1^, 24 h) and stimulated with Ca2^+^ ionophore A23187 (10 µM, 15 min). The cell culture medium was extracted and analyzed by LC‐MS (*n* = 6). D) Representative immunofluorescence staining of CLP (green) and HPGDS (red) in AMs. Nuclei are stained with DAPI (blue). Scale bar: 250 µm (full view), 100 µm (zoom in). E) Expression of CLP and HPGDS in AMs. Western blot analyses were performed on samples from six AM preparations for each condition. F) Cells were sensitized overnight with 500 ng·mL^−1^ anti‐DNP IgE, then stimulated with 100 ng·mL^−1^ DNP‐HSA for 12 h. LC‐MS analysis of LTB_4_ and PGD_2_ present in mast cells (*n* = 6). G) Expression of HPGDS in mast cells. Western blot analyses were performed on samples from six bone marrow‐derived mast cell preparations for each condition. Data shown in (A–C) and (E–G) were presented as mean ± SD. *p* values were assessed using Tukey's multiple comparisons test following two‐way ANOVA for (A–C), (E,F), and Tukey's multiple comparisons test following one‐way ANOVA for (G). *p*< 0.05 was considered statistically significant.

As CLP functions as a scaffold protein for 5‐LO, we examined eicosanoids in bronchoalveolar lavage (BAL). Following the HDM challenge, BAL from *Cotl1* ^−/−^ mice contained lower amounts of leukotriene B_4_ (LTB_4_), but higher levels of PGD_2_ compared to WT mice (**Figure** **3**A). Additionally, prostaglandin E_2_ (PGE_2_) was increased in *Cotl1* ^−/−^ BAL. The PGD_2_ metabolites Δ^12^prostaglandin J_2_ and 15‐deoxy‐Δ^12,14^‐prostaglandin J_2_ were abundant. Δ^12^prostaglandin J_2_ was slightly higher in *Cotl1* ^−/−^ BAL, while 15‐deoxy‐Δ^12,14^‐prostaglandin J_2_ levels were similar between *Cotl1* ^−/−^ mice and WT mice (Figure 3A). BAL from non‐challenged mice contained minimal amounts of all assayed eicosanoids. Enzyme expression related to the production of these eicosanoids was determined in lung tissue by Western blot (Figure 3B). 5‐lipoxygenase activating protein (FLAP) and 5‐LO are crucial in LTB_4_ biosynthesis from arachidonic acid.^[^ ^16^ ^]^ Interestingly, upon the HDM challenge, FLAP levels remained unchanged, and 5‐LO levels decreased in *Cotl1* ^−/−^ mice compared with WT mice (Figure 3B). Hematopoietic prostaglandin D synthase (HPGDS) catalyzes the conversion of PGH_2_ to PGD_2_, and prostaglandin E synthase (mPGES)‐1 and mPGES‐2 convert PGH_2_ to PGE_2_.^[^ ^22^ ^]^ As shown in Figure 3B, both with and without HDM stimulation, the deficiency of CLP significantly increased HPGDS, but no significant effects were found on mPGES‐1 and mPGES‐2. Next, the alveolar macrophages (AMs)transcription factor from WT and *Cotl1* ^−/−^ mice were isolated and cultured to elucidate the role of CLP in PGD_2_ synthesis. Analysis of cell culture supernatants by liquid chromatography‐mass spectrometry (LC‐MS) revealed reduced LTB_4_ and higher levels of PGD_2_ in LPS‐primed and ionophore‐stimulated AMs from *Cotl1* ^−/−^ mice compared with WT mice (Figure 3C). No difference in PGE_2_ production was observed (Figure 3C). In treated cell incubations, the PGD_2_ metabolites Δ^12^prostaglandin J_2_ and 15‐deoxy‐Δ^12,14^‐prostaglandin J_2_ were abundant, though their levels were slightly reduced in cells from *Cotl1* ^−/−^ mice compared with WT mice (Figure 3C). By immunofluorescence, we demonstrated co‐localization of CLP and HPGDS in LPS‐primed AMs from WT mice (Figure 3D). Moreover, *Cotl1* ^−/−^ AMs, both LPS‐primed and controls, expressed more HPGDS compared to WT AMs (Figure 3E). Both mast cells and macrophages may contribute to PGD_2_ biosynthesis in the lungs.^[^ ^23^ ^]^ Intriguingly, when we analyzed PGD_2_ formation and HPGDS expression in DNP‐HSA‐stimulated mast cells derived from the bone marrow of the mice, no differences were found between *Cotl1* ^−/−^ and WT cells (Figure 3F,G). This suggests that the increased PGD_2_ observed in BAL from *Cotl1* ^−/−^ mice mainly originates from AMs rather than mast cells.

### 2.4 Therapeutic Targeting of CLP to Pulmonary Macrophages using AAV‐*Cotl1* Alleviates HDM‐Induced Inflammation

**Figure 4:**
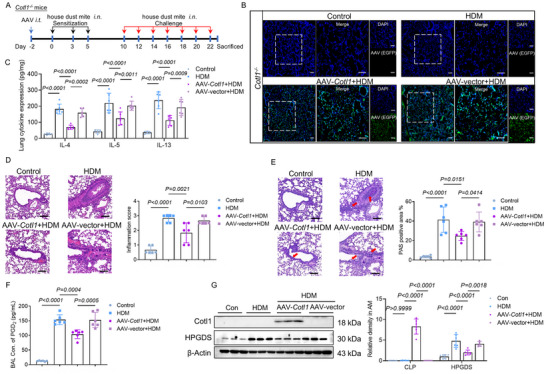
Supplementing CLP in *Cotl1^−/−^* pulmonary macrophages reverses HDM‐induced airway inflammation and PGD_2_ production. A) *Cotl1^−/−^* mice were administered AAV via intratracheal instillation, resulting in elevated CLP expression in pulmonary macrophages. After 2 days, mice were sensitized and challenged intranasally with either HDM (50 µg) or saline on days 0, 3, 5, 10, 12, 14, 16, 18, 20, 22, and were euthanized on day 23. B) Detection of adeno‐associated virus fluorescence (EGFP, green) expression in the *Cotl1^−/−^* lung. Scale bar: 50 µm. C) The levels of Th2 cytokines (IL‐4, IL‐5, and IL‐13) in *Cotl1^−/−^* lung tissue extracts were determined by ELISA (*n* = 6). D,E) Representative *Cotl1^−/−^* lung samples stained with (D) H&E and (E) PAS (*n* = 6). Scale bar: 50 µm. F) LC‐MS analysis of PGD_2_ present in *Cotl1^−/−^* BAL (*n* = 6). G) Expression of CLP and HPGDS in *Cotl1^−/−^* AMs. Western blot analyses were performed on samples from six AM preparations for each condition. Data shown in (C–G) were presented as mean ± SD. *P* values were assessed using Tukey's multiple comparisons test following two‐way ANOVA for (C) and (G), and Tukey's multiple comparisons test following one‐way ANOVA for (D–F). *p*< 0.05 was considered statistically significant.

To therapeutically induce CLP expression in the pulmonary microenvironment, an adeno‐associated viral (AAV) vector specifically targeting mouse pulmonary macrophages (CD68^+^ cells) was engineered to encode *Cotl1* (AAV‐*Cotl1*). *Cotl1^−/−^* mice were treated via the trachea with AAV‐*Cotl1* or a corresponding control virus (AAV‐vector) before the HDM challenge (**Figure** **4**A). The uptake of AAVs was confirmed by the green fluorescent protein (EGFP) observed in mouse lungs (Figure 4B). Importantly, HDM‐challenged *Cotl1^−/−^* mice treated with AAV‐*Cotl1* significantly reduced the levels of Th2 cytokines (IL‐4, IL‐5, and IL‐13) (Figure 4C). Moreover, AAV‐*Cotl1* treatment alleviated inflammation, decreased mucus production, and lowered BAL PGD_2_ levels (Figure 4D–F). Protein expression analysis of mouse AMs showed that AAV‐*Cotl1* treatment decreased the HDM‐mediated HPGDS induction in *Cotl1^−/−^* mice (Figure 4G). Together, these results suggest that CLP induction in pulmonary macrophages may be an effective treatment for HDM‐induced inflammation.

### 2.5 *Cotl1^−/−^* Alveolar Macrophages Exacerbate HDM‐Induced Immune Cell Accumulation and Promote PGD_2_ Receptor CRTH2 Expression

**Figure 5:**
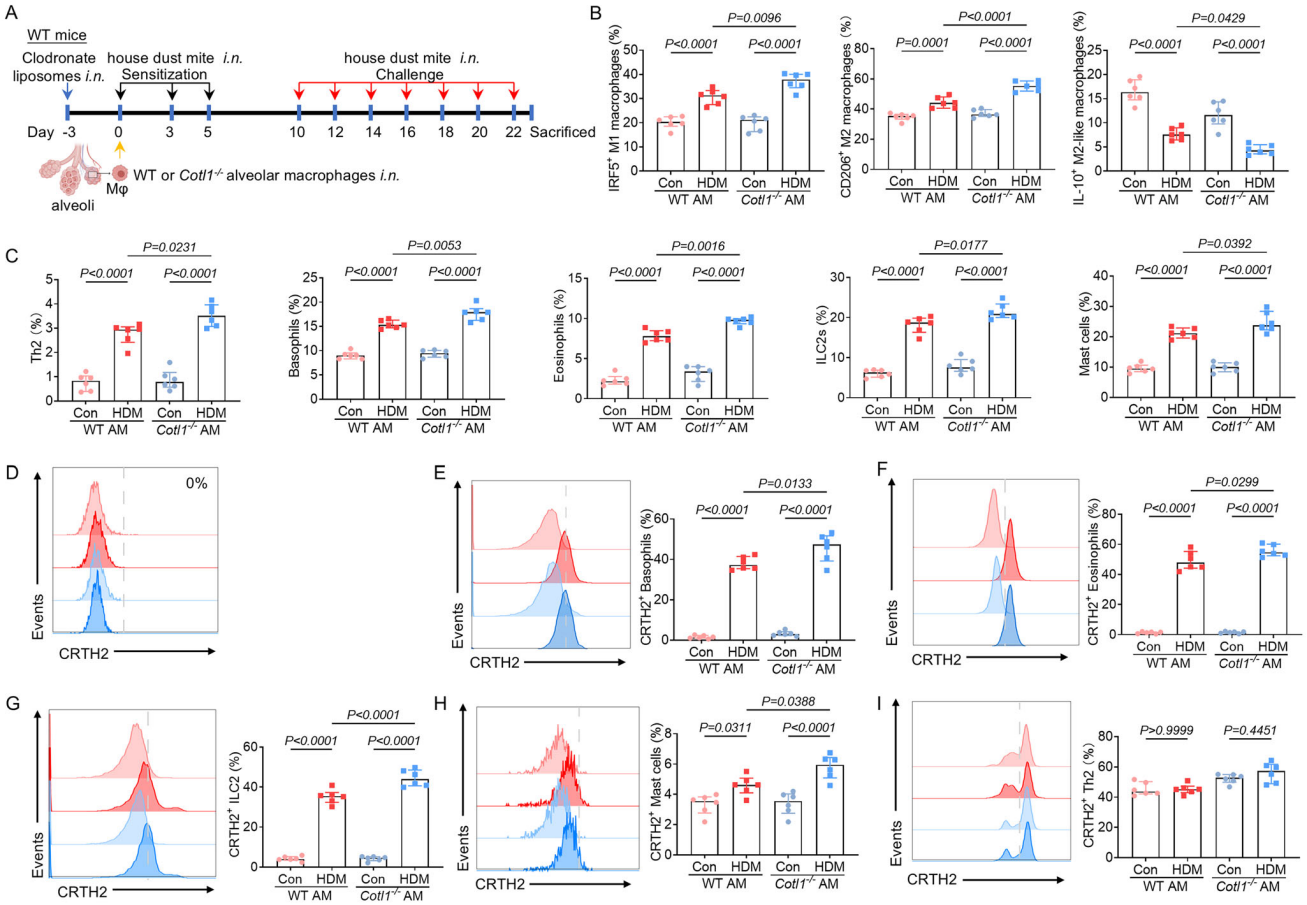
Effect of reconstitution of clodronate‐treated mice with littermate control or *Cotl1* ^−/‐^ alveolar macrophages on HDM‐induced pulmonary immune cell accumulation. A) WT mice received clodronate liposomes to deplete endogenous AMs. After 72 h, the mice were intranasally injected with WT or *Cotl1* ^−/‐^ AMs and treated with HDM. On day 23, mice were sacrificed, and lung tissue single‐cell suspensions were prepared and analyzed by flow cytometry. B) Frequencies of IRF5^+^ M1 macrophages, CD206^+^ M2 macrophages, and IL‐10^+^ M2‐like macrophages among the CD45^+^CD11c^+^CD11b^−^F4/80^+^ population (*n* = 6). C) Frequencies of IL‐4^+^ Th2 cells among the CD4^+^ population, CD49b^+^ basophils among the IgE^+^ population, Siglec‐F^+^ eosinophils among the CD45^+^CD11c^+^ population, Sca‐1^+^KLRG1^+^ ILC2 cells among the CD45^+^CD25^+^Lin^−^ population, and CD117^+^ mast cells among the IgE^+^ population (*n* = 6). D) Frequencies of CRTH2^+^ cells among the macrophage population (*n* = 6). E) Frequencies of CRTH2^+^ cells among the basophil population (*n* = 6). F) Frequencies of CRTH2^+^ cells among the eosinophil population (*n* = 6). G) Frequencies of CRTH2^+^ cells among the ILC2 population (*n* = 6). H) Frequencies of CRTH2^+^ cells among the mast cell population (*n* = 6). I) Frequencies of CRTH2^+^ cells among the Th2 cell population (*n* = 6). Data shown in (B), (C), and (E)‐(I) were presented as median ± interquartile range. *p* values were assessed using Tukey's multiple comparisons test following one‐way ANOVA for (B), (C), and (E–I). *p*< 0.05 was considered statistically significant.

WT mice were intranasally treated with clodronate liposomes to deplete endogenous AMs. After 72 h, the mice were intranasally injected with *Cotl1* ^−/‐^ AMs or WT AMs, followed by HDM priming and challenge (**Figure** **5**A). After the 7th challenge (day 23), the mice were sacrificed, and lung tissue single‐cell suspensions were prepared for immune cell analysis by flow cytometry (Figure 5). Following the HDM challenge, the polarization of alveolar macrophages was further aggravated in *Cotl1^−/−^* AM→WT mice (WT mice received *Cotl1* ^−/−^ AM, Figure 5B). Both in mice receiving WT AMs and *Cotl1* ^−/‐^ AMs, subjected to HDM, relative numbers of Th2 cells, basophils, eosinophils, ILC2, and mast cells increased compared with uninduced mice (Figure 5C). These cell populations were further analyzed for expression of the PGD_2_ receptor CRTH2. Interestingly, macrophages showed no expression of CRTH2 (Figure 5D). However, 40–50% of basophils, eosinophils, and ILC2 from HDM‐challenged lungs expressed CRTH2, compared to less than 3% in control lungs (Figure 5E‐G). For mast cells, CRTH2 expression was low, 5–6% in HDM‐induced cells and 4% in control cells (Figure 5H). The percentage of CRTH2‐expressing basophils, eosinophils, ILC2, and mast cells was all increased in the lung tissue from HDM‐challenged *Cotl1^−/−^* AM→WT mice compared to WT AM→WT mice (Figure 5E–H). For Th2 cells, the high basal expression of CRTH2 was not further increased by HDM challenge (Figure 5I). These results collectively indicate that AMs‐derived PGD_2_ may contribute to disease development via CRTH2 expressed by non‐macrophage cells.

### 2.6 CLP Deficiency‐Induced Exacerbation of Allergic Airway Inflammation Depends on CRTH2

**Figure 6:**
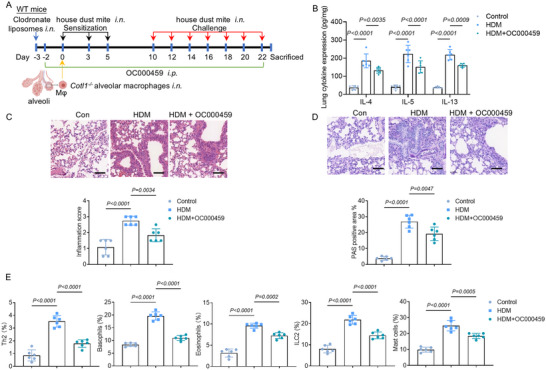
AMs from *Cotl1* ^−/−^ mice confer exacerbated airway inflammation via CRTH2. A) WT mice received clodronate liposomes to deplete endogenous AMs. After 72 h, the mice were intranasally injected with *Cotl1* ^−/‐^ AMs. Mice were challenged with HDM and treated with OC000459. On day 23, mice were euthanized, and lung tissue was analyzed. B) The levels of Th2 cytokines (IL‐4, IL‐5, and IL‐13) in lung tissue extracts were determined by ELISA (*n* = 6). C,D) Representative lung samples stained with (C) H&E and (D) PAS (*n* = 6). Scale bar: 50 µm. E) Frequencies of IL‐4^+^ Th2 cells among the CD4^+^ population, CD49b^+^ basophils among the IgE^+^ population, Siglec‐F^+^ eosinophils among the CD45^+^CD11c^+^ population, Sca‐1^+^KLRG1^+^ ILC2 cells among the CD45^+^CD25^+^Lin^−^ population, and CD117^+^ mast cells among the IgE^+^ population (*n* = 6). Data shown in (B–D) were presented as mean ± SD, and data shown in (E) were presented as median ± interquartile range. *p* values were assessed using Tukey's multiple comparisons test following two‐way ANOVA for (B), and Tukey's multiple comparisons test following one‐way ANOVA for (C–E). *p*< 0.05 was considered statistically significant.

To determine the involvement of PGD_2_ signaling via CRTH2 in exacerbated airway inflammation, mice were treated with CRTH2 antagonist OC000459. WT mice were intranasally administered clodronate liposomes to deplete AMs one day before administration with OC000459 (daily up to day 22). 72 h after clodronate liposome treatment, mice were intranasally injected with *Cotl1* ^−/‐^ AMs. Subsequently, the mice were challenged with HDM (**Figure** **6**A). HDM‐challenged *Cotl1* ^−/−^ AM→WT chimeric mice produced markedly increased Th2 cytokines (Figure 6B) and exhibited severe histomorphological changes (Figure 6C,D). The CRTH2 inhibitor OC000459 partially counteracted these effects. Interestingly, OC000459 was most efficient in reducing the relative number of Th2 cells (Figure 6E). Although CRTH2 expression in mast cells was low, OC000459 also reduced mast cell accumulation in HDM‐challenged lung tissue, suggesting indirect effects on chemotactic factors active on mast cells (Figure 6E). This suggests that HDM‐induced airway inflammation in *Cotl1* ^−/‐^ mice is mediated, at least in part, by PGD_2_ signaling via CRTH2 expressed by non‐macrophage cells.

## 3 Discussion

Our study demonstrates a role of CLP in allergic airway inflammation and potentially in the pathogenesis of asthma. In *Cotl1* ^−/−^ mice exposed to HDM, the composition of alveolar macrophages was altered. Both IRF5^+^ M1 macrophages and CD206^+^ M2 macrophages were upregulated, while IL‐10^+^ M2‐like macrophages were downregulated. This shift was associated with increased PGD_2_ formation and signaling via CRTH2, leading to enhanced immune cell accumulation and exacerbated airway inflammation. These changes were observed after HDM challenge, whereas CLP deficiency had no apparent phenotype effects in unchallenged control mice. A striking finding was that expression of CLP was strongly reduced in WT mice challenged with HDM, seemingly in parallel with the allergic inflammation. Furthermore, overexpression of CLP conferred reduced HDM‐induced inflammation. Thus, loss of functional CLP may contribute to the pathogenesis of allergic asthma.

CLP has multifaceted functions, including modulating the actin cytoskeleton^[^ ^14^ ^]^ and binding to 5‐LO.^[^ ^15^, ^16^, ^17^, ^18^ ^]^ CLP does not directly affect actin polymerization but, as coactosin, is believed to interfere with the capping of actin filaments.^[^ ^24^, ^25^ ^]^ CLP indirectly promotes F‐actin also by protecting it from cofilin‐mediated depolymerization.^[^ ^14^ ^]^ The cytoskeleton is thought to influence the polarization of mouse macrophages through its role in cell geometry.^[^ ^20^ ^]^ Additionally, human M2 macrophages have been shown to depend more on intact actin dynamics compared to M1 macrophages.^[^ ^26^ ^]^ Our findings showed that CLP deficiency promoted IRF5^+^ M1 macrophages and CD206^+^ M2 macrophages while reducing IL‐10^+^ M2‐like macrophages, suggesting distinct roles of the actin cytoskeleton in these macrophage phenotypes.

The metabolism of AA involves a complex network. When one pathway is interrupted, it can lead to the metabolic shunting of AA.^[^ ^27^ ^]^ As expected, LTB_4_ levels in BAL were decreased in *Cotl1* ^−/−^ mice, while unexpectedly, PGD_2_ levels were increased. This increase in PGD_2_ may result from the metabolic shunting of AA to the COX pathway, alongside the increased expression of HPGDS. Interestingly, upregulation of HPGDS in *Cotl1^−/−^* lung tissue as well as in AMs was observed also in the absence of HDM challenge. We also found increased formation of PGD_2_ in ionophore‐incubated AMs from *Cotl1* ^−/−^ mice, but not in mast cells. Likewise, expression of HPGDS was upregulated in lung tissue and AMs, but not in mast cells, from *Cotl1^−/−^* mice.

Mast cells are well‐known to produce PGD_2_ when stimulated by IgE in type 2 inflammation.^[^ ^7^, ^8^, ^9^, ^10^ ^]^ PGD_2_ can also be formed in mouse pulmonary macrophages when stimulated with LPS plus IFN‐γ.^[^ ^23^ ^]^ We found that mouse AMs, when elicited with LPS and activated with ionophore, produced PGE_2_, PGD_2,_ and their cyclopentenone metabolites. Thus, both mast cells and macrophages may contribute to PGD_2_ biosynthesis in the lungs of HDM‐challenged mice. The sensitization and challenge sequence leads to allergic stimulation of mast cells, while pathogen‐associated molecular patterns in HDM stimulate macrophages. The relative contributions of mast cells and macrophages likely change over time, influenced by various factors such as disease types and cell abundance.^[^ ^23^, ^28^, ^29^ ^]^ Our finding that PGD_2_ formation was upregulated in AMs from *Cotl1* ^−/−^ mice, but not in bone marrow‐derived mast cells, suggests that the increase in PGD_2_ observed in *Cotl1* ^−/−^ mice BAL is derived from AMs. This implies that a substantial portion of the PGD_2_ pool in HDM‐challenged mouse lungs is produced by AMs. Regarding further conversions of PGD_2_, it has been reported that macrophages expressing selenoproteins generate PGD_2_ cyclopentenone metabolites.^[^ ^30^ ^]^ We observed relatively high levels of these metabolites, which may indicate PGD_2_ formation, particularly in macrophages. In fact, the sum of PGD_2_ and its metabolites was similar in BAL (and in AM incubations) from both WT and *Cotl1* ^−/−^ mice.

Flow cytometry analysis of pulmonary immune cells revealed distinct surface expression patterns of CRTH2 among various cell types. The frequency of mast cells expressing CRTH2 was low, consistent with the scarce surface presentation of CRTH2 in human mast cells.^[^ ^31^, ^32^ ^]^ CRTH2 expression was absent in alveolar macrophages. For basophils, eosinophils, and ILC2, about 40–50% of these cell populations in HDM‐challenged lungs expressed CRTH2, with higher relative numbers in mice reconstituted with *Cotl1* ^−/−^ AMs. Thus, HDM‐induced allergic inflammation significantly affected the surface presentation of CRTH2 on these cells, a phenomenon not previously described. The Th2‐driving transcription factor GATA3 upregulates CRTH2 expression.^[^ ^33^ ^]^ It has been reported that GATA3 mRNA levels increased in mouse lungs challenged with HDM, providing an explanation for this effect of HDM.^[^ ^34^ ^]^ Early studies indicated that CRTH2 was always expressed on ILC2. However, recent findings have shown that both CRTH2‐positive and CRTH2‐negative ILC2 exist in the lung.^[^ ^35^ ^]^ Our findings suggest that HDM‐induced inflammation upregulates CRTH2 expression in ILC2.

As reported, the effects of PGD_2_ and its cyclopentenone metabolites in inflammation are complex, exhibiting both pro‐inflammatory and anti‐inflammatory properties.^[^ ^36^, ^37^ ^]^ Recently, CRTH2 signaling has been shown to downregulate LPS‐induced nuclear factor kappa‐B activation in murine macrophages.^[^ ^38^ ^]^ A probable reason for these varied observations is that PGD_2_ and its metabolites also act via transcription factor DP1, thromboxane prostanoid, and peroxisome proliferator‐activated receptor‐γ, in addition to CRTH2.^[^ ^7^ ^]^ Our findings support the opinion that animal models confirm a pivotal role for PGD_2_ and CRTH2 in allergic inflammation.^[^ ^7^ ^]^

A limitation of this study is that the role of CLP was investigated in the context of HDM‐induced inflammation. The broader relevance of these findings remains to be established, for example, whether CLP depletion elicits similar effects in other models of allergic airway inflammation, such as ovalbumin‐induced asthma, which warrants further investigation. Additionally, elucidating the molecular mechanisms underlying the upregulation of HPGDS in *Cotl1^−/−^* mice will be important for understanding the broader regulatory network.

## 4 Conclusion

In conclusion, our findings suggest a role for CLP in balancing allergic airway type 2 inflammation in mice. Targeting CLP, or the *Cotl1* gene, could be a potential therapeutic strategy to modulate the immune response in allergic asthma.

## 5 Experimental Section

### Mice

Mice between 6 and 8 weeks of age were bred and housed in a specific pathogen‐free environment at the Experimental Animal Center of Jiangnan University (Jiangsu, China), under controlled temperature and a 12 h light/dark cycle. CLP‐deficient (*Cotl1* ^−/−^) mice (C57BL/6J background) were purchased from the Jackson Laboratory (CA, USA). *Cotl1* ^−/−^ mice and WT mice were acclimatized to laboratory conditions over the course of one week before the experiments. Age‐ and weight‐matched mice were randomly assigned to each group. Mice were sacrificed, and BAL containing AMs and lung tissues were harvested for subsequent assays. All experimental procedures involving mice complied with the ARRIVE guidelines^[^ ^39^ ^]^ and were conducted according to protocols approved by the Institutional Animal Ethics Committee of Jiangnan University (JN. No20200430c1000630[007]).

### Animal Treatments

Mice were intranasally (*i.n*.) primed with HDM (Greer Laboratories, NC, USA) extract (1 µg·µL^−1^, 50 µL) on days 0, 3, and 5. Subsequently, mice were intranasally challenged with HDM (1 µg·µL^−1^, 50 µL) on days 10, 12, 14, 16, 18, 20, and 22. Control mice received saline during both the priming and challenge phases. On day 23, mice were sacrificed and analyzed for airway inflammation. Mice were treated intraperitoneally (*i.p*.) once daily with 1 mg·kg^−1^ of the CRTH2‐specific inhibitor OC000459 (Cayman Chemical, MI, USA), dissolved in dimethyl sulfoxide (DMSO) and diluted in phosphate buffered solution (PBS), starting two days before the first HDM priming and continuing up to day 22. Untreated mice received *i.p*. injections of PBS containing DMSO as a control. Each experimental group consisted of at least 6 mice, as indicated for individual experiments.

### Collection of the BAL Fluid

After euthanasia of the mice, the thoracic cavity was opened, and the pharynx tissue was carefully removed to expose the lungs. An indwelling needle was inserted into the mouth‐trachea‐lungs axis. Subsequently, 1 mL of ice‐cold PBS was injected into the lungs through the indwelling needle, which was then kept in place for 1 min to allow thorough mixing. The PBS solution containing the recovered BAL fluid was then aspirated back into the syringe. This procedure was repeated three times to maximize collection. The collected BAL fluid was transferred to a sterile tube and centrifuged at 350 g for 5 min. The resulting supernatant was collected for LC‐MS analysis.

### Alveolar Macrophage Preparation, Culture, and Treatment

To prepare AM, the BAL fluid was centrifuged at 350 g for 5 min. Cell pellets were then resuspended in incomplete RPMI 1640 medium (Gibco, NY, USA). The pooled cells were counted and seeded into 6‐well plates at a density of 5 × 10^6^ cells per well or into 24‐well plates at a density of 2 × 10^6^ cells per well. After 2 h of incubation, the medium was removed to eliminate non‐adherent cells, followed by washing with RPMI 1640. Complete RPMI 1640 medium was added to the wells, and the cells were cultured at 37 °C in a humidified atmosphere with 5% CO_2_. The extracted cells underwent mycoplasma testing and were confirmed to be free of contamination. To induce an inflammatory state, the cells were primed with 1 µg·mL^−1^ LPS for 24 h. For stimulation of eicosanoid biosynthesis, cells were treated with 10 µM Ca2^+^ ionophore A23187 for 15 min. Supernatants were collected immediately for LC‐MS analysis, and the cells were harvested for Western blot analysis.

### Mast Cell Preparation, Culture, and Treatment

To prepare bone marrow‐derived mast cells (BMMCs), bone marrow cells were cultured in RPMI 1640 medium supplemented with 10% fetal bovine serum (vol·vol^−1^), 10 ng·mL^−1^ IL‐3, and 50 µM 2‐mercaptoethanol for 5–7 weeks.^[^ ^40^ ^]^ The extracted cells underwent mycoplasma testing and were confirmed to be free of contamination. For cell stimulation, the BMMCs were sensitized overnight with 500 ng·mL^−1^ anti‐DNP IgE (Sigma–Aldrich, Shanghai, China) and then stimulated with 100 ng·mL^−1^ DNP‐HSA (Sigma–Aldrich) for 12 h. The cell culture supernatant was collected for LC‐MS analysis, and the cells were collected for Western Blot analysis.

### Bone Marrow Chimeras

The radiation center of the Medical College of Soochow University (Jiangsu, China) was used for irradiation experiments. Unanesthetized mice were restrained in well‐ventilated boxes and exposed to whole‐body γ‐radiation (6 Gy) at a dose rate of 1.00 Gy·min^−1^ at a distance (midpoint) of 85 cm. 4 h after irradiation, mice were injected via the tail vein with bone marrow cells obtained from the femur of either WT or *Cotl1* ^−/−^ mice.

### Adeno‐Associated Virus Treatments

Mice were administered 50 µL of pAAV‐hCD68‐Cotl1‐3xFLAG‐P2A‐EGFP‐WPRE (highly expressing CLP in lung macrophages, AAV‐*Cotl1*, 1 × 10^11^ vg or pAAV‐hCD68‐MCS‐EGFP‐3xFLAG‐WPRE (AAV‐vector) suspension into the trachea using a 22‐G plastic catheter, two days before induction of the allergic asthma model. The AAV‐*Cotl1* and AAV‐vector constructs were synthesized by Obio Company (Shanghai, China).

### AM Depletion and Reconstitution

Mice were sedated by intraperitoneal injection of pentobarbital sodium. Subsequently, 80 µL of a 30% solution of clodronate‐containing liposomes in endotoxin‐free PBS was administered via intranasal instillation. 72 h after clodronate liposome instillation, the AM‐depleted recipient lungs were reconstituted by intranasal administration of 3 × 10^5^ *Cotl1* ^−/−^ AMs or WT AMs.

### Histopathological Analysis

Fresh lung tissue samples were fixed in 4% paraformaldehyde overnight, washed with running water for 2 h, dehydrated with a gradient of ethanol, and then embedded in paraffin. The samples were cut into 5 µm sections using a skiving machine slicer (RM2245, Leica, Hesse, Germany) and stained with H&E and PAS following standard procedures. Lung morphology was evaluated using a Digital Slice Scanner (Pannoramic, 3DHISTECH, Budapest, Hungary). Inflammation scores for perivascular and peribronchial areas were assessed as follows: 1 = single scattered leukocytes; 2 = aggregates less than 10 cells thick; 3 = aggregates ≈10 cells thick; and 4 = numerous coalescing aggregates greater than 10 cells thick. A script in Qupath software was utilized to quantify PAS‐positive areas. Scores were averaged from six randomly selected regions.

### Sample Preparation and LC‐MS Measurement

Eicosanoids were extracted using Oasis HLB solid‐phase extraction columns (Waters, Dublin, Ireland). The columns were preconditioned with 2 mL methanol and equilibrated with 2 mL H_2_O. Methanol (2 volumes) was added to BAL and cell supernatants. Precipitated proteins were removed by centrifugation at 2 000 *g* for 20 min, and the supernatants were collected. The methanol concentration in the supernatants was reduced to 15% with ultrapure water. Then, 0.1% formic acid was added to acidify the sample. Internal standards (Cayman Chemical) were added to each sample. The samples were applied to HLB columns, washed with 2 mL of water containing 0.1% formic acid, 2 mL of water containing 5% methanol and 0.1% formic acid, and then eluted with 2 mL methanol containing 0.1% formic acid followed by 2 mL methyl acetate. The eluates were pooled, solvents were evaporated under a stream of nitrogen, and the samples were reconstituted in 30 µL methanol for LC‐MS analysis.

The samples were analyzed by an LC‐MS system (Q Exactive Plus, Thermo Fisher Scientific; MA, USA) using a C18 reversed‐phase LC column (1.7 µm, 2.1 × 100 mm, Waters), eluted with a gradient composed of water containing 0.1% acetic acid (A) and acetonitrile (B). The gradient program was as follows: 0 min, 65% A, 35% B; 2 min, 65% A, 35% B; 12 min, 15% A, 85% B; 13 min, 15% A, 85% B; 15 min, 65% A, 35% B, at a flow rate was 0.350 mL·min^−1^ and injection volume 5 µL. Analytes were detected using Full MS‐ddMS mode operated in negative mode, with the following source conditions: gas temperature, 300 °C; gas flow, 8.0 L·min^−1^; nebulizer, 30 psi; and ion spray voltage, 4,500 V. LC‐MS/MS parameters for the compounds assayed are shown in **Table** **1**.

**Table 1:** LC‐MS/MS parameters.

Lipid Mediator	Ionization Mode	Retention Time	Parent Ion	Product Ion	Internal Standard	Mass Range
		[min]	[m/z]	[m/z]		
Leukotriene B_4_ (LTB_4_)	[M‐H]^−^	6.83	335.22	195.10	LTB_4_‐d_4_	50–360
Prostaglandin D_2_ (PGD_2_)	[M‐H]^−^	3.91	351.22	189.13	LTB_4_‐d_4_	50–375
Prostaglandin E_2_ (PGE_2_)	[M‐H]^−^	3.45	351.22	271.21	LTB_4_‐d_4_	50–380
Δ^12^prostaglandin J_2_	[M‐H]^−^	5.73	335.22	272.21	LTB_4_‐d_4_	50–365
15‐deoxy‐Δ^12,14^‐prostaglandin J_2_	[M‐H]^−^	8.50	318.21	272.21	LTB_4_‐d_4_	50–345
Standard‐LTB_4_	[M‐H]^−^	6.83	335.22	195.10		50–360
Standard‐PGD_2_	[M‐H]^−^	3.88	351.22	189.13		50–375
Standard‐PGE2	[M‐H]^−^	3.45	351.22	271.21		50–380
Standard‐Δ^12^prostaglandin J_2_	[M‐H]^−^	5.73	335.22	272.21		50–365
Standard‐15‐deoxy‐Δ^12,14^‐prostaglandin J_2_	[M‐H]^−^	8.50	318.21	272.21		50–345

### Immunoblotting

Lung tissue samples were homogenized in ice‐cold lysis buffer RIPA containing protease and phosphatase inhibitors. Protein concentrations were determined by the BCA Protein Assay Kit (Beyotime, Shanghai, China). AMs were lysed and homogenized, and a 10 000 *g* supernatant was prepared. Equal amounts of protein (typically 30 µg) were electrophoretically separated in sodium dodecyl sulfate‐polyacrylamide gels and transferred onto the nitrocellulose filter membrane (Merck KGaA, Hesse, Germany). The membranes were blocked with 5% w·v^−1^ nonfat dry milk in TBS‐T for 1 h at room temperature, followed by overnight incubation with appropriately diluted primary antibodies at 4 °C. After washing, membranes were probed with secondary peroxidase‐labeled antibodies for 2 h at room temperature. Antibodies for CLP (10781‐1‐AP), HPGDS (22522‐1‐AP), mPGES‐2 (10881‐1‐AP), CRTH2 (25264‐1‐AP), and β‐actin (60008‐1‐lg) were purchased from Proteintech (Hubei, China). Antibodies for 5‐LO (ab39347), FLAP (ab85227), and mPGES‐1(ab180589) were purchased from Abcam (Cambridge, UK). Protein bands were visualized using enhanced chemiluminescence with a ChemiDoc Imager (Bio‐Rad Laboratories, CA, USA). Densitometric analyses of protein expression by Western blot were performed using Image J software (CA, USA).

### Flow Cytometry

Freshly harvested lung tissue samples were digested in PBS containing 1 mg·mL^−1^ collagenase‐4 (Sangon, Shanghai, China) at 37 °C for 15 min. Tissue was then homogenized using gentle MACS Dissociators (Miltenyi Biotec; Bergisch Gladbach, Germany). After removing red blood cells by lysis buffer (FMS‐RBC050, Fcmacs, Jiangsu, China), samples were filtered through a 75 µm filter screen with PBS. Single‐cell suspensions were prepared, and surface staining was performed after blocking Fc receptors with FcR blocking reagent (130‐092‐575, Miltenyi Biotec) for 5 min at 4 °C, followed by incubation for 15 min at room temperature in PBS with the following antibodies: PE/Cy7 anti‐mouse CD45 (103114), Alexa Fluor 488 anti‐mouse CD11c (117313), PE anti‐mouse IL‐10 (505 008), PE anti‐mouse IL‐4 (504104), Brilliant Violet 421 anti‐mouse Sca‐1 (108127), Brilliant Violet 421 anti‐mouse F4/80 (123137), Brilliant Violet 650 anti‐mouse CD25 (102037), PE anti‐mouse CD206 (141705), FITC anti‐mouse Lineage cocktail (133301), PE anti‐mouse KLRG1 (138407), PE anti‐mouse CD49b (108907) and PE anti‐mouse CD117 (105807) from BioLegend (CA, USA). PE anti‐mouse IRF5 (IC8447P) from R&D (MN, USA). PE‐Cy7 anti‐mouse CD4 (552775), PE anti‐mouse Siglec‐F (552126), and Brilliant Violet 421 anti‐mouse IgE (564207) from BD Biosciences (NJ, USA). APC anti‐mouse CD11b (17‐0112‐81) Alexa Fluor 647 anti‐mouse CRTH2 (51‐2941‐82) from eBioscience (CA, USA).

For flow cytometry staining of intracellular cytokines (IL‐4 and IL‐10), lung single‐cell suspensions were stimulated with 20 ng·mL^−1^ phorbol 12‐myristate 13‐acetate, 1 µg·mL^−1^ ionomycin and 10 µg·mL^−1^ brefeldin A at 37 °C for 4 h. Cells were then treated with Fixation Buffer for 15 min at room temperature, washed, and incubated with anti‐IL‐4 or IL‐10 antibodies in the presence of Permeabilization Buffer for 15 min at room temperature. The protocol followed the manufacturer's instructions (88‐8824‐00, Intracellular Fixation & Permeabilization Buffer Set, eBioscience). Flow cytometry staining for the transcription factor IRF5 followed the instructions provided with the Transcription Factor Staining Buffer Set (00‐5523‐00, eBioscience).

Gating methods of fluorescence‐activated cell analysis were programmed as follows: IRF5^+^ M1 macrophages, CD45^+^CD11c^+^CD11b^−^F4/80^+^IRF5^+^; CD206^+^ M2 macrophages, CD45^+^CD11c^+^CD11b^−^F4/80^+^CD206^+^; IL‐10^+^ M2‐like macrophages, CD45^+^CD11c^+^CD11b^−^F4/80^+^IL‐10^+^; Th2 cells, CD4^+^IL‐4^+^; eosinophils, CD45^+^CD11c^+^Siglec‐F^+^; basophils, IgE^+^CD49b^+^; group 2 innate lymphoid cells (ILC2), CD45^+^CD25^+^Lin^−^KLRG1^+^Sca‐1^+^; mast cells, IgE^+^CD117^+^. In all experiments, dead cells were excluded using Fixable Viability Dye (65‐0865‐14, eBioscience). Stained cells were analyzed on an Attune NxT flow cytometer (Thermo Fisher Scientific), and data were analyzed using FlowJo 10.8.1 software (BD Biosciences).

### Immunofluorescence and Confocal Microscopy

Alveolar macrophages adhering to polylysine‐coated glass slides were fixed in 4% formaldehyde, permeabilized with 0.1% Triton X‐100, and blocked with 10% normal goat serum. The cells were then incubated with primary antibodies: rabbit anti‐HPGDS antibody or mouse anti‐CLP antibody, followed by appropriate secondary antibodies: Alexa Fluor 594‐labeled donkey anti‐rabbit IgG (H+L) highly cross‐adsorbed secondary antibody (A32754, Thermo Fisher Scientific) for HPGDS, or Alexa Fluor 488‐labeled goat anti‐mouse IgG1 cross‐adsorbed secondary antibody (A‐21121, Thermo Fisher Scientific) for Cotl1. Nuclei were counterstained with DAPI (4′,6‐diamidino‐2‐phenylindole). Confocal microscopy was performed using a Zeiss LSM700 confocal microscope equipped with plan‐Apochromat 63X/1.4 and plan‐Neofluor 40X/1.3 oil immersion lenses.

### Statistical Analysis

Statistically significant differences among three or more groups were determined using a one‐way or two‐way ANOVA with Tukey's *post hoc* test, while differences between two groups were assessed using an unpaired two‐tailed *t*‐test. Data were expressed as mean ± standard deviation (SD). Flow cytometry data were expressed as median ± interquartile range. *p* value< 0.05 was considered statistically significant. Statistical analyses were performed using GraphPad Prism 9.5.0 software (GraphPad, CA, USA).

## Conflict of Interest

The authors declare no conflict of interest.

## Author Contributions

L.L.P., Z.R., B.L., W.L., and Y.L. contributed equally to this work. B.S. was the Co‐senior author. L.‐L.P., Z.R., B.L., W.L., and Y.L. performed experiments and analyzed data, with general assistance from Q.Y., H.L., X.D., H.T., and H.Z., B.S., and O.R. provided intellectual inputs and contributed to project planning. JS and OR designed and interpreted experiments. J.S., O.R., Y.L., Z.R., B.L., and L.‐L.P. wrote the paper. All authors critically reviewed and finally approved the manuscript.

## Data Availability

The authors declare that all data supporting the results of this study were available within the article. Further information and requests for resources and reagents should be directed to and will be fulfilled by the lead contact, Prof. Jia Sun (jiasun@jiangnan.edu.cn).
